# EzArray: A web-based highly automated Affymetrix expression array data management and analysis system

**DOI:** 10.1186/1471-2105-9-46

**Published:** 2008-01-24

**Authors:** Yuerong Zhu, Yuelin Zhu, Wei Xu

**Affiliations:** 1 Research and Development, BioInfoRx, Inc., Middleton, WI 53562, USA; 2 Department of Oncology, Georgetown University, Washington DC, 20057, USA; 3 Department of Oncology, University of Wisconsin-Madison, Madison, WI 53706, USA

## Abstract

**Background:**

Though microarray experiments are very popular in life science research, managing and analyzing microarray data are still challenging tasks for many biologists. Most microarray programs require users to have sophisticated knowledge of mathematics, statistics and computer skills for usage. With accumulating microarray data deposited in public databases, easy-to-use programs to re-analyze previously published microarray data are in high demand.

**Results:**

EzArray is a web-based Affymetrix expression array data management and analysis system for researchers who need to organize microarray data efficiently and get data analyzed instantly. EzArray organizes microarray data into projects that can be analyzed online with predefined or custom procedures. EzArray performs data preprocessing and detection of differentially expressed genes with statistical methods. All analysis procedures are optimized and highly automated so that even novice users with limited pre-knowledge of microarray data analysis can complete initial analysis quickly. Since all input files, analysis parameters, and executed scripts can be downloaded, EzArray provides maximum reproducibility for each analysis. In addition, EzArray integrates with Gene Expression Omnibus (GEO) and allows instantaneous re-analysis of published array data.

**Conclusion:**

EzArray is a novel Affymetrix expression array data analysis and sharing system. EzArray provides easy-to-use tools for re-analyzing published microarray data and will help both novice and experienced users perform initial analysis of their microarray data from the location of data storage. We believe EzArray will be a useful system for facilities with microarray services and laboratories with multiple members involved in microarray data analysis. EzArray is freely available from .

## Background

One of the major problems that life science researchers have to cope with is the management of huge amounts of data, which is ever increasing with advances in robotics and microarray technologies. More and more laboratories have begun adopting Structured Query Language (SQL)-based relational databases, such as Oracle and MySQL, to solve life sciences data management problems. Another major problem in life sciences is secure and efficient data sharing, especially when the data is in large scale. A common temporary solution is using shared folders on the internet or intranet; however, this option provides minimal data security and is accompanied by difficulties in associating information with files. Therefore, novel, easy-to-use, and powerful data management and sharing systems are needed in the life sciences.

Though microarray-based experiments are becoming popular in life science research, microarray data management and analysis are still challenging tasks for many biologists. Researchers often use manual or custom developed systems for microarray data management and analysis, which are limited by developers' knowledge of computer sciences, biostatistics, and life sciences. Commercial products for microarray data analysis are being released, such as GeneSpring GX from Agilent Technlogies, Santa Clara, CA 95051 and GeneSifter from VizX Labs, Seattle, WA 98119. However, these products are often complicated, expensive, and/or lack data sharing capabilities. For biostatisticians and experienced analysts, R language [1] and Bioconductor [2] are the main microarray data analysis tools. R is a widely used open source language for statistical computing and graphics. Bioconductor, which is primarily based on the R programming language, is an open development software project for the analysis and comprehension of genomic data. Currently, hundreds of Bioconductor packages have been developed, providing comprehensive functionalities for all aspects of microarray data analysis. For example, *affy *is often used for low-level analysis of Affymetrix GeneChip data, while *multtest *is useful in detecting differentially expressed genes. Currently, web-based systems based on R and Bioconductor packages are being developed, such as CARMAweb [3], MAGMA [4], GEPAS [5], Asterias [6], ArrayPipe [7], MIDAW [8], RACE [9], WebArray [10], and Expression Profiler [11]. While these systems have made microarray data analysis much easier for experienced users, much improvement is needed to further automate the common data analysis processes so that they can be readily accessible to novice users.

In order to provide an easy-to-use microarray system for researchers with little pre-knowledge of microarray data analysis as well as experienced analysts, we have designed a web-based system, EzArray, based on the most recent web technologies, R, and Bioconductor. EzArray is intended to provide: 1) a centralized location to store original microarray data with security; 2) an easy and secure way to share raw data and analyze results among team members; 3) a highly automated data analysis system for instant on-line data analysis; 4) an expandable system to integrate new data management and analysis tools.

## Implementation

To implement EzArray, we adopted the popular database and web application software bundle LAMP which refers to Linux operating system, Apache web server, MySQL database, PHP programming language. Selecting these technologies is mainly based on features such as low technical requirements for webmasters, programmers, and end users, open source, rapid application development, low total cost of ownership, and extremely large resources for free application source codes. In addition, we heavily incorporated Ajax (*Asynchronous Javascript And XML*) technologies to increase the system's interactivity, speed, functionality, and usability.

**Figure 1:**
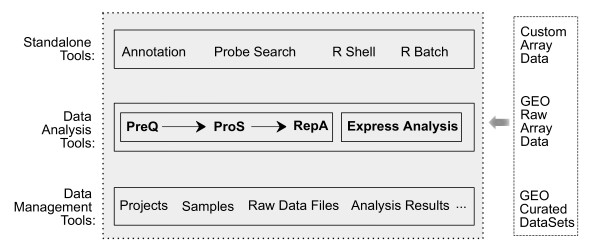
**EzArray is an Affymetrix expression array data management and analysis system**. EzArray can be used to manage and share data including projects, samples, raw array data files, and analysis results. EzArray includes three highly automated and seamlessly integrated data analysis programs named PreQ for data preprocessing and quality assessment, ProS for data processing and statistical testing, and RepA for report generating and gene annotation. Express Analysis is a one-step data analysis tool that covers all processing procedures in PreQ, ProS, and RepA. Microarray data can be from users' experiments (Custom Array Data), published raw array data (deposited CEL supplementary files in GEO), or GEO curated DataSets (GDS records). In addition, a number of standalone tools have been included in EzArray, including tools for gene annotation, array probe search, R shell for interactive execution of R scripts, and R batch for batch execution of R scripts.

On EzArray server, PHP scripts deal with communication between users and the server, dynamically generate R scripts based on user input, execute R scripts in the background, and parse R output and present results to end users as HTML webpages. User information, data files, project information and analysis results are stored in database and server file system. EzArray comes with a web-based file management tool (My Files) and a request job management tool (Job List). On the client end, users logically follow these steps: register, logon, create or join a user group, create projects, import sample information and upload microarray data, submit analysis requests and browse results. The analysis tools (PreQ, ProS, and RepA) can be used in orders as shown in Figure 1. Users can perform each type of analysis multiple times with modified parameters.

**Figure 2:**
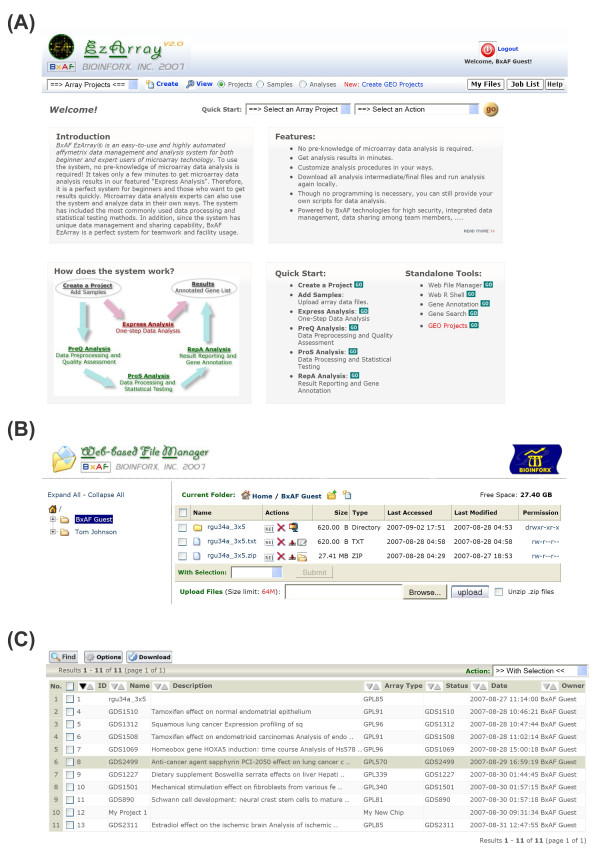
**EzArray is a web-based system implemented with advanced web technologies**. (A) A screenshot of the EzArray homepage. The most important navigation tool in EzArray is the menu bar under the EzArray logo. However, users can also use the Quick Start pull-down menus, the hyper-linked diagram, or the Quick Start links to get started. (B) Explorer-like online file management and group-based file sharing. While file owners have full control of their files (*e.g*. create new folders and files, rename or delete existing folders and files), group members can read, copy, and download others' files, but are not allowed to make changes. (C) Full-featured project search and browse tool. Users can browse project information page by page, update the viewing options, perform advanced searches, and download data in spreadsheet format. Similar tools are available for managing sample information and analysis results (not shown).

Figure 2A shows a screenshot of the EzArray (version 2) homepage, Figure 2B shows a screenshot of the integrated file management tool, and Figure 2C shows a screenshot of the project browsing and searching tools.

## Results

### EzArray system architecture

We propose that an ideal microarray system should be easy-to-use for all levels of users, have minimal software and hardware requirements for installation and usage, have data privacy for each user but also allow data sharing with others, be flexible to integrate custom tools, and provide maximum data analysis reproducibility. Based on these ideas, we developed EzArray (Figure 1 and Figure 2) in the open source application development environment LAMP that consists of Linux operating system, Apache web server, MySQL database, and PHP programming language. The combination of these technologies has become popular because of its low acquisition cost and because of the ubiquity of its components. We release EzArray under the GNU General Public License, providing end users maximal freedom in taking advantage of the EzArray source codes.

EzArray is a multi-user system with web interfaces. All users must first register, and their accounts will become active upon approval by system administrators. User login information is stored in MySQL database with encryption, making EzArray a highly secure system.

### Microarray data management and sharing in EzArray

In EzArray, data are either stored in MySQL database or on the server as files. Microarray data are organized by projects, and a user can create unlimited projects. Currently, only minimal project information is required, including Affymetrix array chip type, a brief project description, and optional project details. Collecting minimal project information allows users to get started quickly. In each project, only one array chip type is allowed. Besides Affymetrix chip type names such as hug133plus2, users can also enter Gene Expression Omnibus (GEO) [12] platform names such as GPL570. The array sample information and the CEL files generated from Affymetrix GeneChip Operating System can be added one-by-one or imported into a project in batch. In EzArray data analysis can be performed with all samples in a project or just a few selected samples.

EzArray projects, samples, and analysis results entered by a user may be shared with group members in read-only mode (Figure 2C for project management). However, EzArray administrators can adjust data sharing methods by changing the settings in the configuration file.

### Microarray data analysis procedures in EzArray

Microarray data analysis in EzArray is highly automated such that even novice users can perform initial analysis and get results instantly. Experienced users can also use the system with full control of the analysis procedures. In general, microarray data analysis is performed step-by-step with these programs: PreQ – preprocessing, normalization, and quality control plots; ProS – statistical procedures for detecting differentially expressed genes; and RepA – gene annotation and linking to public databases (Figure 1).

PreQ reads the raw Affymetrix expression array data files (CEL files) and completes all necessary data preprocessing, including data background correction, data normalization, correction for non-specific binding, and summarization where the measured probe intensities are averaged to one expression value per probe set (Table 1). There are four pre-defined data preprocessing methods (RMA, MAS5, dChip, and GCRMA) and one custom method. RMA method uses robust multichip average (rma) algorithm [13,14] for background correction. MAS5 method adopts the Affymetrix MAS5 algorithm [15-18]. dChip uses a special Li-Wong summarization algorithm [16,19] that is a model-based approach, allowing pooling of information across multiple arrays and automatic probe selection to handle cross-hybridization and image contamination. GCRMA uses the background correction method gcrma [16] that takes probe sequence information into calculation. The custom method in PreQ allows users to select specific preprocessing algorithms for each type of processes. Table 1 shows a summary of algorithms used in each PreQ method. Detailed comparison of the different Affymetrix preprocessing algorithms can be found in reference [20]. The *affy *package from Bioconductor is used in PreQ for most data preprocessing tasks. In addition to data preprocessing, PreQ is a convenient tool to assess data quality since it generates many quality assessment plots. The current EzArray version includes histoplots of the intensity data, *affyPLM *plots that fit probe level models to array data, RNA digestion plots in which ordered probes are used to detect possible RNA degradation, *simpleaffy *QC plots that provide access to many of the standard QC functions recommended for Affymetrix arrays, MA plots that are widely used to compare two intensity measurements, scatter plots that show the correlation of cell intensity across arrays, and boxplots that help users evaluate the differences in the distributions of intensities across arrays. Reference [21] studied microarray data quality assessments and provides a good summary of quality assessment plots.

**Table 1:** Summary of EzArray microarray data preprocessing methods

***Methods***	***Background Correction***	***Normalization***	***PM correction***	***Summarization***
RMA	rma	quantiles	pmonly	medianpolish
MAS5	mas	mas	mas	mas
dChip		invariantset	pmonly	liwong
GCRMA	gcrma	quantiles	pmonly	medianpolish
Custom Methods:	rma rma2 mas gcrma-eb gcrma-mle	quantiles quantiles.robust loess contrast constant invariantset qspline vsn	pmonly mas subtractmm	medianpolish avdiff liwong mas playerout rlm

The predefined methods RMA, MAS5, and GCRMA are named mainly based on the background correction algorithms, while dChip is named based the unique summarization algorithm. Custom method allows users to specify algorithms for background correction, normalization, PM correction, and summarization. Please review references [15, 16, 33, 34] and references therein for descriptions of these algorithms and comparisons among them.

The ProS program in EzArray processes array data and detects differentially expressed genes with different methods based on the number of sample groups and replicates in each sample group (Table 2). When there are only two sample groups, *e.g*. experimental condition verse control condition, and a small number of biological replicates (less than 3) in each sample group, ProS simply uses fold changes to detect differentially expressed genes. When a sufficient number of arrays are used in microarray experiments, *e.g*. three or more replicates in each sample group, various statistical tests can be used to detect differentially expressed genes. Available statistical tests in the current EzArray version include two-sample Welch t-test, two-sample t-test, standardized rank sum Wilcoxon test, paired t-test, F-test, Block F-test, and more. EzArray allows the users to select a number of multiple hypothesis testing methods to control error rate. References [22,23] provide summaries of multiple hypothesis testing methods and their applications in microarray experiments. The main Bioconductor package used in ProS program is *multtest*.

**Table 2:** EzArray has built-in algorithms that help users select statistical testing methods based on the number of sample groups and replicates

**Sample Groups**	**Sample Replicates**	**Basic Statistical Method**	**Multiple Testing Procedure**	**Gene (feature) Limit Options**	**Main result files**
1	Any	None	None	None	None

2	< 3 in either group	• Average values of each gene (feature) within group • Calculate fold change of each gene between groups	None	1. Fold Change 2. Total number of genes 3. User gene list	• Gene list • Fold changes • Expression values • Heatmap of top genes
	
	≥3 in both groups	• two-sample Welch t-test (unequal variances) • two-sample t-test (equal variances) • standardized rank sum Wilcoxon test • paired t-test • Options for Raw/Nominal p-value calculation: - *Parametric* - *Permutation* - Options for Side/Rejection Region: abs, upper, lower	• Bonferroni single-step FWER • Holm step-down FWER • Hochberg step-up FWER • Sidak single-step FWER • Sidak step-down FWER • Benjamini & Yekutieli step-up FDR • Benjamini & Hochberg step-up FDR – selected • Storey q-value single-step pFDR • Westfall & Young maxT permutation FWER • Westfall & Young minP permutation FWER	• Fold Change • Limit to - *Total number of genes* - *adjusted p-values* - *raw p-values* - *test statistics* • *User gene list*	• Gene list • Fold changes • Statistic • Raw p-values • Adjusted p-values • Expression values

≥3	≤3 in any group	• Calculate percentile of standard deviation (SD) of each gene cross all samples • Select genes by a SD percentile cutoff		• Standard Deviation (SD) • Total number of genes • User gene list	• Gene names • Standard Deviation • Expression values
	
	≥3 in all groups	• F-test • Block F-test • Options for Raw/Nominal p-value calculation: - Parametric - Permutation - Options for Side/Rejection Region: abs, upper, lower	• Bonferroni single-step FWER • Holm step-down FWER • Hochberg step-up FWER • Sidak single-step FWER • Sidak step-down FWER • Benjamini & Yekutieli step-up FDR – selected • Benjamini & Hochberg step-up FDR • Storey q-value single-step pFDR • Westfall & Young maxT permutation FWER • Westfall & Young minP permutation FWER	• Total number of genes • User gene list	• Gene names • Statistic value • Expression values

While users can specify the statistical testing methods and parameters for the analysis, EzArray has built-in logics to select methods and parameters in the default modes. In general: 1) RMA is used to pre-process data; 2) If there is only one sample group, no analysis is performed; 3) If there are two groups and one or both of them has less than three replicates, then a fold change cut-off is applied, and the system returns the top genes (both up and down regulated, up to 100 in total) with 1.8 or higher fold change; 4) If there are three or more groups and one or more of them have less three replicates, then a Standard Deviation (SD) cut-off is applied, and the system returns the top genes (both up and down regulated, up to 100 in total) with SD within 90%; 5) If there are two groups and both of them have three or more replicates, then t test and Benjamini & Hochberg step-up FDR (BH) are applied, and the system returns the top 100 genes ordered by adjusted p-values, unadjusted p-values, and test statistics and with fold change >= 1.8; 6) If there are three or more groups and all groups have three or more replicates, then F-test and BH are applied, and the system returns the top 100 genes ordered by adjusted p-values, unadjusted p-values, and test statistics.

The RepA program is a web interface of the Bioconductor package *annaffy *that produces compact HTML and text reports including experimental data and hyperlinks to many online databases. RepA makes the results more understandable for biologists. While the RepA program is tightly linked to the ProS program, EzArray also provides two *annaffy*-based standalone tools that allow users to annotate genes from a list of probe names or search for probes based on gene annotation information.

Though these three analysis programs are tightly connected and are normally used in sequential order, experienced users can use them individually or combine them with their own analysis scripts. For example, users can first use the highly automated PreQ program to complete data preprocessing and generate necessary quality assessment plots. Then, based on the initial results, users can download and revise the analysis scripts for further analysis.

**Figure 3:**
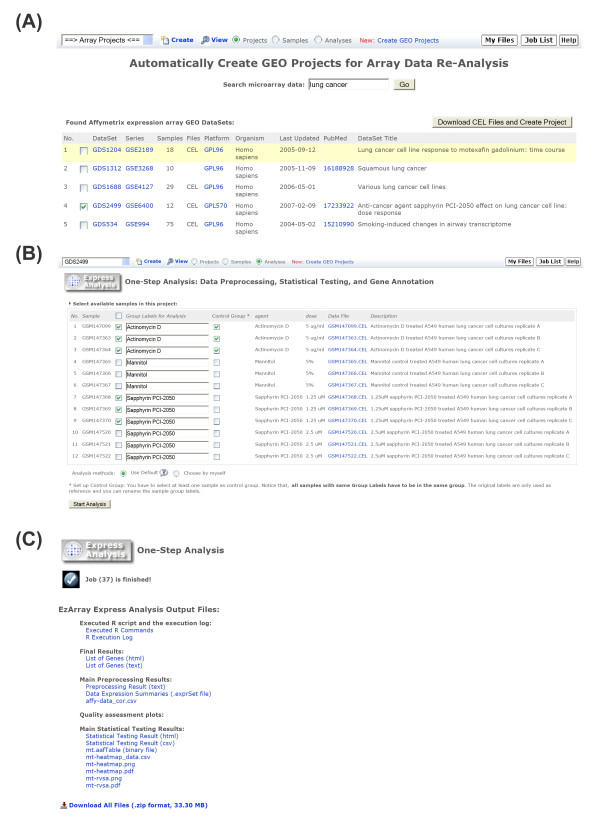
**Express Analysis is a fully automated microarray data analysis program**. (A) Users can search for GEO DataSets, download CEL supplementary files, and create EzArray projects to analyze previously published data. The sample information is automatically populated in the project based on the subset information stored in GEO GDS records. (B) Selecting samples to start new Express Analysis. While in most cases, default analysis methods and parameters can be used directly due to our built-in logics, experienced users have options to select methods and enter specific analysis parameters. Once the analysis is started, a pop-up window will appear showing currently running jobs. On the pop-up window, users can stop running jobs, remove failed jobs, or review finished jobs. In addition, users do not have to wait for results; instead, they can bookmark the page and come back later to review the results. (C) Example execution results from a run of Express Analysis with data shown in (B). The resulting files, including executed scripts and execution logs, are classified, listed, hyper-linked, and compressed in one file for easy downloading.

In order to make our microarray data analysis system practically useful for users with less knowledge of biostatistics and also make it a convenient system for experienced users, we further optimized and integrated our three-step analysis programs into a one-step data analysis program called Express Analysis. With Express Analysis, if users select the optimized settings (Table 2), they can obtain a list of differentially expressed genes with annotation in just a single click. Figure 3 shows the screenshots of EzArray Express Analysis from searching GEO databases (Figure 3A), selecting samples (Figure 3B), and finally, obtaining analysis results (Figure 3C). Again, experienced users can select "custom" methods to tune data analysis parameters and select desired analysis algorithms. Express Analysis in EzArray represents the most automated microarray data analysis program currently released.

### Re-analysis of previously published microarray data

EzArray can be used to re-analyze previously published Affymetrix expression array data that were deposited in GEO, the main gene expression/molecular abundance repository. Due to rapid advances of microarray data analysis technologies and specialized foci of previous microarray researchers, it will be of significance to re-analyze some published microarray data. In addition, with increasingly accumulated microarray data in GEO, it becomes possible to study some new research projects using deposited microarray data from different laboratories. For these purposes, we have added a very convenient tool in EzArray allowing users to search GEO microarray data, automatically download array data, and create new projects with automatically populated sample information (Figure 3).

EzArray contains tools to retrieve the descriptive information of all GEO records, including Platforms, Series, Samples, and DataSets. EzArray also contains a simple search form allowing users to search for DataSets (Figure 3A). The search results include a list of DataSets with links to GEO website. Users can select one or more DataSets from the list and create projects for data re-analysis. EzArray downloads the corresponding DataSet files automatically from the GEO website.

Analysis of GEO microarray data may be started from raw microarray data (*e.g*. .CEL files) or pre-processed expression value data originally submitted by previous authors. In the first case, if authors have submitted .CEL files to GEO, EzArray will automatically download these supplementary files and link them to the newly created projects. Then users can perform data analysis with EzArray programs as previously stated. In the second case, regardless of whether authors submitted raw microarray data or not, EzArray retrieves curated datasets from GEO, extracts the dataset information, and stores them in the project, which can be readily used to perform data re-analysis with program ProS directly without performing data pre-processing with PreQ.

## Discussion

EzArray is a web-based Affymetrix expression array data management and analysis system implemented in an open source environment. Since the same technologies are often used to build database-powered websites, EzArray can be easily integrated with users' existing websites. In summary, EzArray takes advantages of modern web technologies, provides multiple user support, has group-based data sharing capabilities, contains tools for highly automated data analysis, and has user-friendly interfaces. These features distinguish EzArray from most other standalone and web-based microarray programs.

Most microarray data analysis tools have been implemented as Bioconductor R packages that run from the command line or have simple point-and-click graphic interfaces. Both R packages *limma *[24] and *affy *offer R users a command-line interface to state-of-the-art microarray data analysis techniques. The R packages *affylmGUI *[25] and *webbioc *offer simple point-and-click interfaces to many of the *limma *and *affy *functions. It seems these programs simply analyze data instead of providing comprehensive data management capabilities.

Recently, more and more web-based microarray systems have been developed. MAGMA [4] is a Java-based web application that provides a simple and intuitive interface to identify differentially expressed genes from two-channel microarray data. MAGMA does not support databases, and the results are file-based. Though MAGMA provides for each user a separate workspace for storing and analyzing microarray data, MAGMA lacks tools for data sharing among users. Similar to EzArray, MAGMA automatically generates R-scripts that document the entire data processing steps. However, EzArray takes it further by allowing the users to download all input and output files together with R-scripts. This guarantees the user to regenerate all results in his local R installation. In terms of data analysis, MAGMA does not contain the gene annotation step and the results are tab-delimited text files and graphic plot files. The RepA program in EzArray generates HTML webpages with hyperlinks to public life science databases. In addition, compared to EzArray, MAGMA does not include algorithms to automatically select data analysis methods and parameters, and therefore, the analysis process is less automated. GEPAS [5] has been designed to provide an intuitive web-based interface that offers diverse analysis options from data preprocessing to gene selection, gene clustering, gene annotation, and more. Instead of taking advantages of existing R and Bioconductor packages, GEPAS has incorporated many newly developed programs written in 'C' languages. The web interfaces of GEPAS are Perl CGIs. The most recent version of GEPAS (v4.0) has included very simple tools for user registration as well as data file browsing. In addition, due to the abundance of novel programs and low level of automation in data analysis, using GEPAS requires in-depth knowledge of the system and many microarray data analysis algorithms. Asterias [6] is an open source and web-based suite for the analysis of gene expression and aCGH data. Asterias is the only web-based application that uses parallel computing. Asterias also takes advantages of many R and Bioconductor packages including *limma*. The web interfaces of Asterias are mostly written in Python. Though a few applications in Asterias support MySQL database, Asterias does not contain any tools for user or data management. The input data to all applications are plain text files that are uploaded "on the fly" during analysis. The web application CARMAweb [3] was implemented in Java based on J2EE (Java 2 Enterprise Edition) software technology. It supports Affymetrix GeneChips, spotted two-color microarrays and Applied Biosystems (ABI) microarrays. CARMAweb has a simple user management tool that guarantees password protected access to the user's data and analysis results. All user data are stored as files in the user data directory. Currently, CARMAweb does not support databases and group-based data sharing. WebArray [10] is another microarray system implemented with technologies similar to those used in EzArray (WebArray used Python instead of PHP programming language). WebArray provides a user-friendly interface for accessing a wide range of key functions of *limma *and other Bioconductor packages. WebArray is an excellent free open source software system for microarray analysis that can be used by an average biologist after moderate training. Nevertheless, WebArray has limited capabilities in data management and data sharing. WebArray is not project-oriented and all data are stored as files in one user data directory. Though WebArray allows users to download output files (tab-delimited text files and graphic plots), it does not allow downloading of executed R scripts. When compared to these web microarray systems, EzArray features much more intuitive user interfaces, more powerful data management capabilities, and significantly higher levels of automation in the analysis processes.

EzArray was designed to be operating system-independent due to the cross-platform features of Apache and PHP. EzArray is also expected to be database platform-independent due to the adoption of a database abstraction library ADOdb [26] that supports most SQL-based databases. This provides the flexibility for end users to select convenient operating systems and database servers. So far, we have fully tested EzArray on the Linux operating system (Fedora 7) with MySQL database, and we are planning to test EzArray on other operating systems with various databases.

The current version of EzArray stores only minimal experimental information. We are planning to develop new database tables and corresponding web interfaces for storing MIAME [27]-compliant microarray data.

Due to the modular structures and open source features of EzArray, extensions or new functionalities can be rapidly implemented on top of EzArray. We have already started designing web-based tools for analyzing Agilent and Nimblegen microarray data. Even with Affymetrix expression data, our analysis procedures can be further improved. For example, for data with two sample groups and just a few replicates per group, the current version of EzArray simply uses Fold Changes to select differentially expression genes. In next EzArray version, we plan to enhance the data analysis procedures with more established algorithms and programs, such as *limma*, SAM [28-30], and EBArrays [31,32].

## Conclusion

EzArray is an Affymetrix expression array data management, analysis, and sharing system. Besides tools for users to organize their own microarray data online and perform instant data analysis, EzArray contains tools for re-analyzing previously published microarray data deposited in GEO. EzArray can not only help novice users perform initial analysis of their microarray data, but also allow experienced users to perform custom analysis from the location of data storage. In summary, EzArray will be a useful system for facilities with microarray services and laboratories with multiple members involved in microarray projects.

## Availability and requirements

EzArray is released under General Public License and can be freely used at website . To install EzArray locally, users need to set up a Linux server running Apache and MySQL. Experienced users may be able to install EzArray on Mac or Windows operating systems with different database servers. Recent versions of R and Bioconductor should be pre-installed and properly configured.

## Authors' contributions

YuerongZ developed the main idea, coded the majority of EzArray, and drafted the manuscript. YuelinZ was involved in system and statistics design, coded the majority of R scripts, performed overall program debugging and testing, and was involved in revising the manuscript. WX provided helpful discussion and performed software tests. She was also involved in revising the manuscript. All authors read and approved the final manuscript.
